# Development and validation of an evidence-based otorhinolaryngological care protocol for patients with achondroplasia: a pilot study

**DOI:** 10.1016/j.bjorl.2026.101942

**Published:** 2026-10-31

**Authors:** Cynthia Moreno, Marcial Galera

**Affiliations:** a Universidade Federal de Mato Grosso (UFMT), Hospital Universitário Júlio Müller (HUJM), Head and Neck Unit, Cuiabá, MT, Brazil; b Universidade Federal de São Paulo (UNIFESP), São Paulo, SP, Brazil; c Universidade Federal de Mato Grosso (UFMT), Pediatrics Department, Cuiabá, MT, Brazil

**Keywords:** Achondroplasia, Otorhinolaryngological manifestations, Integrative review, Care protocol

## Abstract

**Objective:**

To develop an evidence-based otorhinolaryngological care protocol for patients with achondroplasia, and to evaluate its content validity and clinical applicability through expert assessment and pilot testing.

**Methods:**

This methodological study was conducted in three stages. Initially, this evidence-based protocol was developed based on a literature review of the most prevalent otorhinolaryngological manifestations in patients with achondroplasia, utilizing the PRISMA guidelines. guidelines for systematic reviews. Second, content validation was performed by a panel of three specialists (a pediatrician, a geneticist, and an otorhinolaryngologist) and conducted in two consecutive rounds using a structured questionnaire on the Google Forms platform. The questionnaire employed the Likert scale and the CVI (Content Validity Index). Third, a pilot application of the protocol was conducted in four patients aged 2–10 years, with confirmed diagnosis of achondroplasia, followed at a tertiary outpatient clinic, to assess feasibility, clarity, and applicability in clinical practice.

**Results:**

The protocol provides systematic and comprehensive data collection, including clinical history, otorhinolaryngological examination, and complementary assessments. Content validation demonstrated high agreement among experts, with a CVI of 0.91, indicating excellent clarity, relevance and applicability. At pilot phase, the protocol proved to be feasible and easy to apply, with an average application time of 34-minutes.

**Conclusion:**

The proposed protocol is an evidence-based, feasible, and clinically applicable tool that supports standardized otorhinolaryngological assessment in patients with achondroplasia. Its implementation may contribute to early identification of complications, guiding appropriate therapeutic decision-making, improving the quality of care and providing a rich database for future research.

**Level of Evidence 4/5:**

This methodological study involved the development of an evidence-based clinical protocol, expert content validation, and pilot application in a clinical setting, without a comparative or interventional design.

## Highlights

• Evidence-based ENT protocol for achondroplasia.
• Evidence-based otorhinolaryngological manifestations in patients with achondroplasia.
• Content validation by expert panel (pediatrician, geneticist, and otolaryngologist).
• The protocol was applied in a clinical setting, confirming its clinical feasibility.
• Structured care improves assessment, diagnosis, treatment and future research.

## Introduction

Achondroplasia (ACH) is the most common disproportionate short stature skeletal dysplasia; its estimated worldwide prevalence at birth is 4.6 per 100,000,^1^ with incidence at birth of 1:10,000 to 1:30,000.^2^ In Latin American countries, the prevalence ranges from 0.26 to 0.45 per 10.000 births.^1^

The main physical characteristics include short stature; rhizomelia; macrocephaly; midface hypoplasia; small chest; thoracolumbar kyphosis; lumbar hyperlordosis; limited elbow extension; short fingers and trident-shaped hands; hypermobile hips and knees; medial bowing of the legs and hypotonia.^3^

Craniofacial abnormalities in achondroplasia, including premature fusion of the skull base synchondroses, maxillary hypoplasia, cranial foramina stenosis and cervicomedullary compression, compromise both the spatial relationships and tone of the upper airway, as well as auditory functioning. Associated with factors such as macroglossia, pharyngeal hypotonia, high arched palate and depressed nasal bridge, further contribute to the otorhinolaryngological manifestations of the disease.^4^, ^5^, ^6^, ^7^, ^8^, ^9^, ^10^, ^11^, ^12^

A systematic review with meta-analysis on the impact of achondroplasia in Latin American patients, which studied outcomes such as mortality, physical comorbidities, humanistic and economic outcomes, reports that ENT disorders accounted for 21.42% of the physical comorbidities. Furthermore, in a pooled analysis of 230 patients, 5 (2.17%) underwent adenotonsillectomy, 75 (32.6%) had mildly to moderately increased apnea index, 75 (32.6%) had desaturations during sleep and apnea, and 32% pooled proportion of ENT disorders (hearing loss, recurrent otitis media, need for surgical treatment, speech development delay, hypotonia, adenoid hypertrophy, snoring, tonsillectomy and tympanic membrane perforation) was observed, demonstrating the importance of otorhinolaryngological symptoms in these patients.^1^ Another study demonstrates, in published case series, that clinically significant obstructive apnea is reported in about one-third of individuals of all ages with achondroplasia. Middle ear dysfunction is present in 50%–70% of cases, which results in approximately 50% of individuals requiring ventilation tube insertion at some point in life, and estimates of hearing loss frequency range from 38% to 60% in achondroplasias population.^3^

Currently, there are no specific protocols described in the literature for otorhinolaryngological management of patients with achondroplasia, and care of these patients is based on generalist practices.

Therefore, the objective of this study was to develop, validate the content, and conduct a pilot application of an otorhinolaryngological care protocol for patients with achondroplasia in a clinical setting.

The clinical protocol was developed based on evidence, followed structured stages, including the identification of care gaps, a literature systematic review, and structuring with a definition of theme, title, presentation, introduction, objective, target population, included approaches, research questions, implementation indicators, methods for evidence search, references, and documentation, as recommended in the literature.^13^^,^^14^

The development and implementation of a specific otorhinolaryngological care protocol for patients with achondroplasia aims to standardize follow up of disease manifestations, prevent complications from untreated dysfunctions, and increase the accuracy of therapeutic indications.

## Methods

This methodological study, conducted between April 2024 and February 2026, was developed in three stages. The first comprised an integrative literature review^15^^,^^16^ on otorhinolaryngological manifestations in achondroplasia, guided by the research question: “What are the main otorhinolaryngological manifestations in patients with achondroplasia described in worldwide literature?” Searches were conducted in the MEDLINE, BVS (Biblioteca Virtual em Saúde), LILACS, and Cochrane Library databases, using Medical Subject Headings (MeSH/DeCs) descriptors combined with Boolean operators.

Articles published between 2010 and 2025, in Portuguese, English or Spanish, with open access to full text were included, studies selection was conducted independently by two reviewers according to inclusion and exclusion criteria according to PRISMA guidelines^17^ and evaluated using the Joana Briggs Institute critical appraisal tools,^18^, ^19^, ^20^, ^21^, ^22^, ^23^, ^24^, ^25^, ^26^ excluding articles with a score below 70%. Data analysis was qualitative with thematic categorization and descriptive statistical analysis, profiling the manifestations to support the protocol development. The validated Brazilian Portuguese version of the Pediatric Sleep Questionnaire (PSQ) was used in the protocol for obstructive sleep apnea screening, given its good sensitivity and specificity relative to polysomnography.^27^

The second stage involved content validation by a panel of experts composed of a pediatrician, a geneticist, and an otorhinolaryngologist. This was conducted in two consecutive rounds using a structured Likert-scale questionnaire, in which each item in each session received ratings from 1 to 4, corresponding to the following options: (1) Strongly disagree; (2) Partially disagree; (3) Partially agree; (4) Strongly agree. Assessments were performed using the Google Forms.^28^ Based on the results of the second round, the CVI (Content Validity Index) was calculated: first the CVI of each item was calculated, obtained by the ratio between the sum of the item scores assigned by the three experts and the maximum possible score, which in this case is 12, and subsequently the arithmetic mean between of all items scores was performed to determine the overall CVI of the protocol. After each round, the experts’ contributions were analyzed and incorporated into the document, resulting in its final version.

In the third stage, a pilot application of protocol was conducted in a convenience sample of patients with a confirmed diagnosis of achondroplasia, followed at the medical genetics’ outpatient clinic of the Hospital Universitário Júlio Muller, Universidade Federal de Mato Grosso (HUUFMT), aged 2–14 years, to assess feasibility, clarity, and applicability in clinical practice.

## Results

### Literature review

The literature review included 27 studies,^1^^,^^2^^,^^4^, ^5^, ^6^, ^7^^,^^10^^,^^29^, ^30^, ^31^, ^32^, ^33^, ^34^, ^35^, ^36^, ^37^, ^38^, ^39^, ^40^, ^41^, ^42^, ^43^, ^44^, ^45^, ^46^, ^47^, ^48^ comprising 15 primary and 12 secondary studies. Review articles were retained for their informative value, with no risk of duplication, as they were not used to estimate prevalence. The selection process was completed using a PRISMA (Preferred Reporting Items for Systematic Reviews and Meta-Analyses^17^ flow diagram, presented in Fig. 1.

**Fig. 1:**
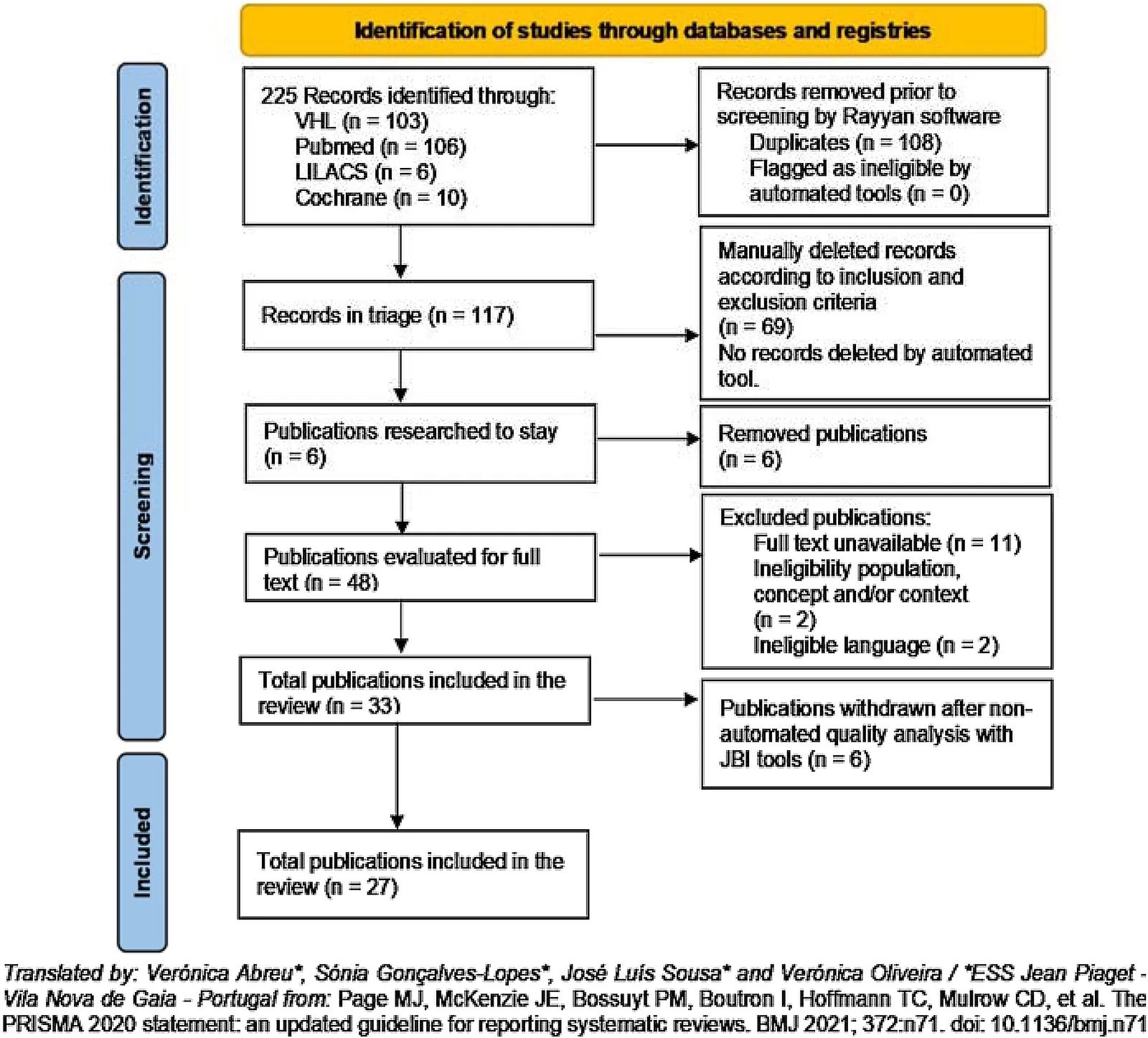
Study selection process flow diagram adapted from PRISMA Statement recommendations.^17^ Source: Author.

Regarding the characterization of the selected studies, geographical distribution showed a significant concentration of scientific output in Europe (39.26%), Asia (23.69%), and North America (22.22%); Latin America accounted for 10.81% of studies, with Brazil representing 5.25%. Among the 27 studies, 12 (44.44%) focused on the pediatric population, 5 (18.52%) on the adult population, and 10 (37.04%) included participants from both age groups.

An analysis of the absolute citation frequency of each otorhinolaryngological manifestation per article was performed across the 27 studies, with the findings presented in Fig. 2.

**Fig. 2:**
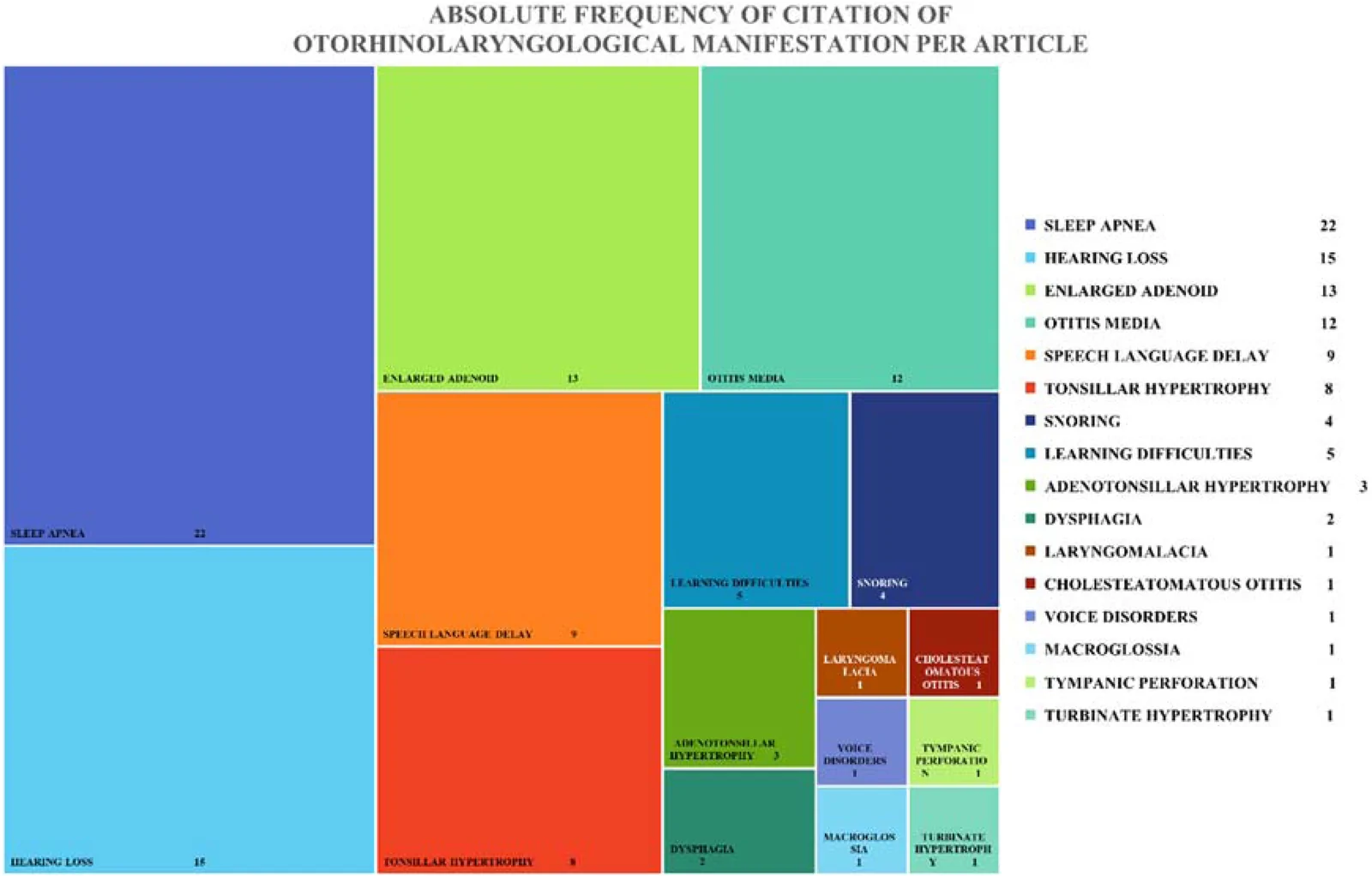
Treemap chart with absolute frequency of otorhinolaryngological manifestations citation in prevalence studies included in the review. Source: Author. Note: Absolute frequency of citation for each manifestation was used since the same study may cite more than one manifestation.

An analysis of the absolute citation frequency of each otorhinolaryngological manifestation per article was also conducted based on 11 prevalence studies^5^^,^^7^^,^^29^^,^^30^^,^^32^^,^^34^^,^^36^^,^^37^^,^^40^^,^^41^^,^^44^ and the results are presented in Fig. 3.

**Fig. 3:**
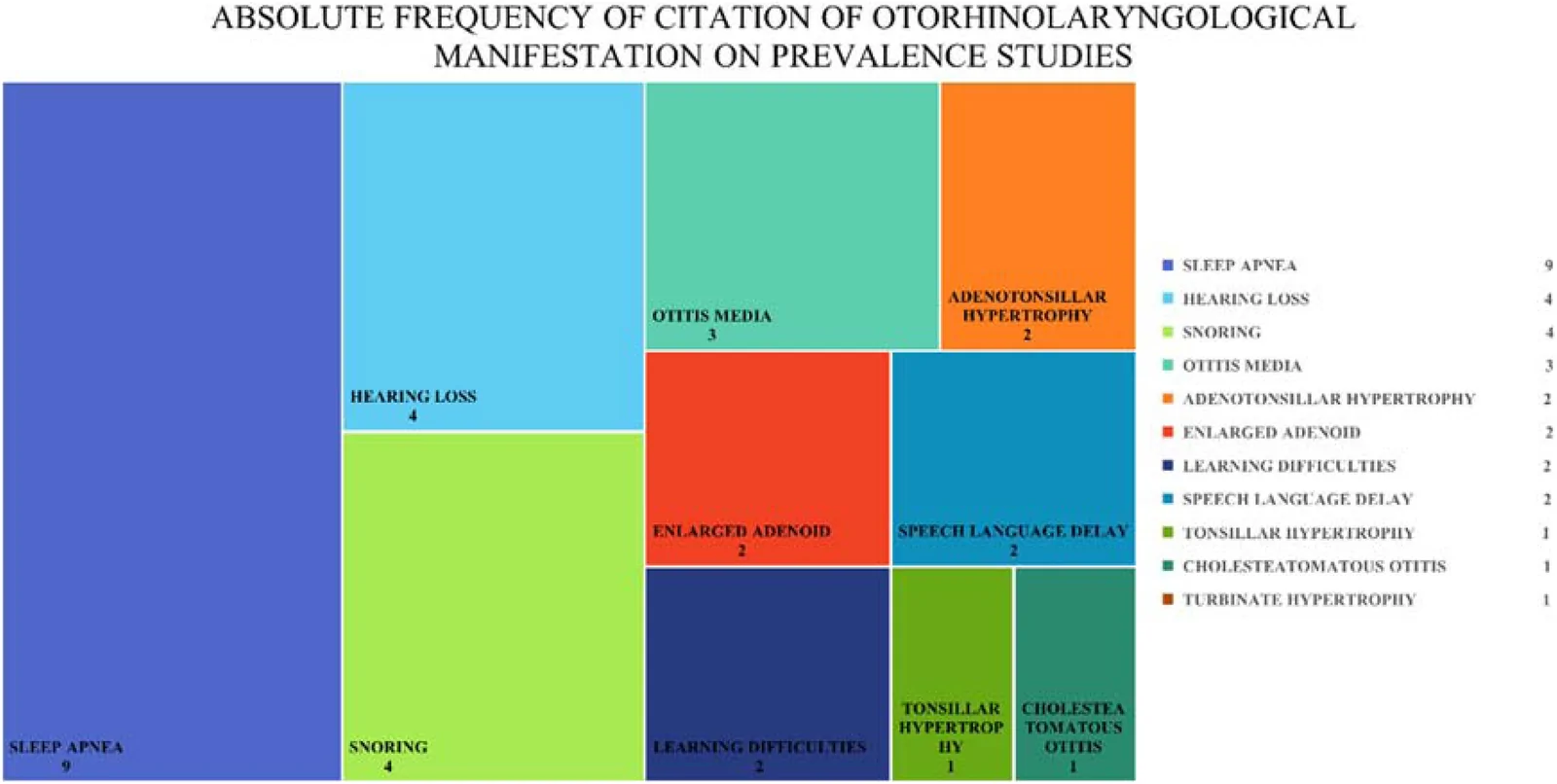
Treemap chart with absolute frequency of otorhinolaryngological manifestations citation in prevalence studies included in the review. Source: Author. Note: Absolute frequency of citation for each otorhinolaryngological manifestation was used since the same study may cite more than one manifestation.

A synthesis of the range of each otorhinolaryngological manifestation reported in the 11 prevalence studies reviewed is summarized in Table 1.

**Table 1:** Synthesis of prevalences of otorhinolaryngological manifestations in the 11 prevalence studies included in the review.

Otorhinolaryngological Manifestations	Reference
Otitis media (72.9%)	^5^
Hearing loss (43.8%)	
Enlarged adenoid (25.7%)	
Cholesteatomatous otitis media (2.9%)	
OSA (30.2%)	^29^
Snoring (94.3%)	
Otitis media (54.7%)	
Hearing loss (9.4%)	
Enlarged adenoid (56.6%)	
Speech and language delay (40.8%)	
Learning difficulties (14.7%)	
OSA (70.37%)	^7^
Adenotonsillar hypertrophy (62.9%)	
Hearing loss (53%)	^30^
Conductive (63%)	
Mixed (33%)	
Sensorioneural (4%)	
OSA (38.4%)	^41^
OSA (59%)	^40^
Snoring (86%)	
Mild OSA (13.6%)	^36^
Moderate OSA (13.6%)	
Severe OSA (72.7%)	
Mixed apnea (37%)	
Adenotonsillar hypertrophy (63.7%)	
OSA (53.8%)	^37^
Otitis media (37%)	
Middle ear dysfunction (54%)	
Hearing loss (11%)	
Speech delay (41%)	
OSA (56%)	^44^
Mild (29%)	
Moderate (9%)	
Severe (16%)	
Learning difficulties (34%)	
OSA (33%)	^32^
Snoring (77%)	
OSA (54.3%)	^34^
Mild (28.26%)	
Moderate (15.21%)	
Severe (10.86%)	
Snoring (69.9%)	

The results demonstrate that respiratory manifestations, such as snoring and obstructive sleep apnea, and otological manifestations, such as otitis media and hearing loss, constitute the most prevalent otorhinolaryngological complications in achondroplasia.

### Content validation

Regarding the results of the protocol evaluation by the expert panel, consensus was reached on the adequacy and applicability, with high agreement among the experts, allowing for final validation of the instrument content with an overall Content Validity Index (CVI) of 0.94. Table 2 presents the protocol evaluation items as rated by the experts, using the Likert scale and the Content Validity Index (CVI).

**Table 2:** Evaluation of protocol items by the expert panel according to the Likert scale and the Content Validity Index (CVI).

Question	Pediatric evaluator	Geneticist evaluator	ENT evaluator	CVI (Content Validity Index)
The items on this form are relevant for the initial otolaryngological evaluation of patients with achondroplasia.	4	4	4	1
The requested information is clear and logically organized for completion in clinical practice.	4	4	4	1
The length (number of items) of this form is adequate for routine outpatient care (neither excessive nor insufficient).	4	3	3	0.83
I believe this form is feasible to fill out in most of the services where I work/am familiar.	4	3	4	0.91
Questions about pregnancy are relevant for the otolaryngological follow-up of patients with achondroplasia.	4	3	4	0.83
The items in this section allow you to properly identify relevant information about your pregnancy.	4	4	4	1
The wording of the items is clear and easy for the professional who will be filling them out to understand.	4	4	4	1
The amount of information requested in this section is adequate for routine use.	4	4	3	0.91
Questions about childbirth are relevant for the otolaryngological follow-up of patients with achondroplasia.	4	3	4	0.91
The items in this section allow for the proper identification of complications during childbirth.	4	4	4	1
The wording of the items is clear and easy for the professional who will be filling them out to understand.	4	4	4	1
The amount of information requested in this section is adequate for routine use.	4	4	3	0.91
Questions about newborns are relevant for the otolaryngological follow-up of patients with achondroplasia.	4	4	4	1
The items in this section allow you to properly identify relevant information about the newborn.	4	4	3	0.91
The wording of the items is clear and easy for the professional who will be filling them out to understand.	4	4	4	1
The amount of information requested in this section is adequate for routine use.	4	4	4	1
Questions regarding the diagnosis of achondroplasia are relevant for the otolaryngological follow-up of patients with achondroplasia.	4	3	4	0.91
The items in this section allow you to properly identify relevant information about the newborn.	4	4	3	0.91
The wording of the items is clear and easy for the professional who will be filling them out to understand.	4	4	4	1
The amount of information requested in this section is adequate for routine use.	4	3	3	0.83
Questions about growth milestones are relevant for the otolaryngological follow-up of patients with achondroplasia.	4	3	4	0.91
The items in this section allow for the proper identification of relevant feeding difficulties (swallowing, food transition).	4	3	4	0.91
The wording of the items is clear and easy for the professional who will be filling them out to understand.	4	4	4	1
The amount of information requested in this section is adequate for routine use.	4	4	4	1
Questions about motor development are relevant for the otolaryngological follow-up of patients with achondroplasia.	4	4	4	1
The items in this section allow for the proper identification of relevant feeding difficulties (swallowing, food transition).	4	3	4	0.91
The wording of the items is clear and easy for the professional who will be filling them out to understand.	4	4	4	1
The amount of information requested in this section is adequate for routine use.	4	3	4	0.91
Questions about breastfeeding and the introduction of solid foods are relevant for the otolaryngological follow-up of patients with achondroplasia.	4	4	4	1
The items in this section allow for the proper identification of relevant feeding difficulties (swallowing, food transition).	4	3	4	0.91
The wording of the items is clear and easy for the professional who will be filling them out to understand.	4	3	3	0.83
The amount of information requested in this section is adequate for routine use.	4	3	4	0.91
The items in this section are suitable for tracking speech, language, and learning changes in patients with achondroplasia.	4	4	4	1
The questions allow for the early identification of signs of delays or communication disorders.	4	4	4	1
Information regarding language, speech, schooling/daycare, and learning difficulties is relevant to the context of the protocol.	4	3	4	0.91
The language used in this section is clear and understandable for professionals in the field.	4	4	3	0.91
The items in this section comprehensively and pertinently cover the main otorhinolaryngological signs and symptoms relevant to achondroplasia.	4	3	4	0.91
The structure of the questions facilitates the identification of the main otorhinolaryngological signs and symptoms.	4	3	4	0.91
Questions about otorhinolaryngological signs and symptoms are appropriate.	4	3	4	0.91
This section is feasible to implement in practice (number of items, clarity, completion time).	4	4	3	0.91
The items in this section adequately address the main relevant anatomical and functional changes in patients with achondroplasia.	4	4	4	1
The registration method (YES/NO/NOTES and specific fields) is clear and functional.	4	3	4	0.91
This section promotes a systematic and comprehensive assessment of the otolaryngological examination.	4	3	4	0.91
The number of items is appropriate for the needs of an outpatient clinic, without making the examination excessively time-consuming.	4	2	4	0.83
The inclusion of complete audiometry, videonasopharyngolaryngoscopy, and polysomnography is relevant for the follow-up of patients with achondroplasia.	4	4	4	1
The available fields are sufficient to record the most relevant findings from the examinations.	4	3	4	0.91
The recording method (normal/altered + observations) is simple and suitable for clinical practice.	4	3	4	0.91
This section promotes the integration of complementary tests into the clinical reasoning of the protocol.	4	4	4	1
The use of PSQ is appropriate and well-founded for screening sleep-related breathing disorders in children with achondroplasia (27).	4	4	4	1
The way PSQ is integrated into the protocol makes it easier for professionals to apply and interpret (27).	4	4	4	1
The guidelines regarding the cutoff point (a result greater than 9 “YES” responses) are clear.	4	4	4	1
I believe it is feasible to apply the PSQ (Product Quality Assessment) to the daily service routines in the areas where I work/am familiar (27).	4	3	4	0.91
The annual follow-up form is suitable for monitoring the otorhinolaryngological clinical evolution of patients with achondroplasia over time.	4	3	4	0.91
The structure of this form maintains consistency with the initial assessment, facilitating comparison between consultations.	4	4	4	1
The number of items and the method of recording are feasible for annual use in service practice.	4	4	3	0.91
This form facilitates the organization of longitudinal follow-up and the identification of relevant clinical changes.	4	3	4	0.91
Is the protocol applicable to real-world clinical practice?	4	4	4	1
Does the protocol contribute to improving care for patients with achondroplasia?	4	4	4	1
Is the protocol ready for clinical use?	4	3	4	0.91
**0.94**				

After content validation, the final version of the protocol includes two assessment forms, one for the first consultation and another for otorhinolaryngologic follow-up visits.

The first form includes information on pregnancy, delivery, newborn, achondroplasia diagnosis, growth milestones, motor development milestones, feeding, speech and language development, otorhinolaryngological signs and symptoms, otorhinolaryngological physical examination, and complementary ENT tests, as well as the Pediatric Sleep Questionnaire (PSQ) Brazilian Portuguese version for obstructive sleep apnea (OSA) screening, with good sensitivity and specificity compared to polysomnography.^27^ If the PSQ score is ≥9, this is suggestive of OSA, and specific management will be discussed case by case. The second form includes information on intercurrent events and evolution since the previous consultation, ongoing treatments, growth milestones, motor development milestones, feeding, speech and language development, otorhinolaryngological signs and symptoms, otorhinolaryngological physical examination, complementary ENT tests, and the Pediatric Sleep Questionnaire (PSQ).^27^ The final protocol structure is presented in Table 3.

**Table 3:** Otorhinolaryngological care protocol for patients with achondroplasia.

Initial outpatient care form (first consultation with ent)																
**Patient's name:**																
**Medical Record Number: Date of Birth: / /**																
**Age: Sex: () F () M**																
**Name of the person in charge:**																
**Telephone:**																
**Medical History Reports: ____________________________________________________________________________________________________________________________________________________________________________________________________________________________________________________________________________________________________________________________________________________________________________________________________________________________________**																
**PREGNANCY INFORMATION**																
		YES		NO									Observations			
Prenatal care																
Alcoholism																
Smoking																
Other drugs																
Infection																
Familiar history of congenital anomaly																
Changes in gestational ultrasound																
Prenatal diagnosis of achondroplasia													If so, when?			
Fetal distress																
**INFORMATION ABOUT CHILDBIRTH**																
Type of delivery		Vaginal () Cesarean ()														
Gestational age																
Maternal age																
**NEWBORN INFORMATION**																
Weight																
Length																
Head circumference																
Apgar																
		YES		NO									Observations			
Perinatal complications													If so, which ones?			
Phenotypic alterations													If so, which ones?			
Congenital infection													Observations			
**ACHONDROPLASIA DIAGNOSIS**																
Age																
Diagnostic method(s)		Clinical () Radiological () Genomic ()														
		YES		NO								Observations				
Currently undergoing specific treatment												Medication and onset				
Other treatments																
**GROWTH MILESTONES** Hoover-Fong et al.^49^																
		Measurement		Percentile									Observations			
Weight																
Head circumference																
Height																
Weight/Height																
**MOTOR DEVELOPMENT** Ireland et al.^50^																
		Age		Percentile							Observations					
Cervical control (months)																
Sat with support (months)																
Sat up without support (months)																
Crawled (months)																
Independent march (months)																
**FEEDING**																
BREASTFEEDING		Exclusive breastfeeding () until ___ months. Formula () Mixed () up to ___ months.														
Notes:																
Feeding introduction		without difficulty ()				with difficulty ()										
Observations (need for support, semi-sitting, hypotonia, acceptance of solid foods/tongue protrusion reflex, food refusal, cough, choking).																
**SPEECH AND LANGUAGE DEVELOPMENT** Ireland et al.^50^																
		Age		Percentile							Observations					
Social smile																
Babbling																
First words																
First sentences																
Understands verbal commands.																
Observations (characteristics, form of communication):																
		YES		NO					Observations							
Speech therapy									Observations							
Is he/she in school or daycare?									Stage?							
Learning difficulties									Observations							
**OTORHINOLARYNGOLOGICAL SIGNS AND SYMPTOMS**																
		YES		NO					Observations							
Nasal obstruction																
Respiratory noise																
Mouth breathing																
Snoring																
Tonsillitis																
Otitis									Otorrhea?							
Rhinosinusitis																
Hearing loss																
Salivar control																
Clinical suspicion of Obstructive Sleep Apnea (OSA)																
Dysphagia									() Liquid () Paste () Solid							
Choking									() Liquid () Paste () Solid							
Previous aspiration pneumonia																
Otorhinolaryngological Surgery									Which?							
**OTORHINOLARYNGOLOGICAL EXAMINATION**																
		YES		NO					Observations							
Oropharyngeal abnormalities																
Palate abnormalities																
Dental abnormalities																
Language abnormalities																
Anterior rhinoscopy																
Otoscopy																
Facial skeletal abnormalities (Midface hypoplasia, micrognathia, retrognathia)																
Skull base / possible narrowing of the foramen magnum (clinical suspicion)																
Other facial morphological abnormalities																
**ADDITIONAL TESTS IN OTOLARYNGOLOGY**																
**Exam**		**Normal**		**Changed**					**Unrealized**							
Pure-tone audiometry																
Impedance audiometry																
Auditory Evoked Potentials																
Videonasopharyngolaryngoscopy																
Polysomnography																
**PSQ QUESTIONNAIRE PORTUGUESE VERSION – BRAZIL** Martins et al.^27^**APPLICATION DATE:**																
**While your child sleeps:**					**Yes**			**No**								**Don't know**
A1. Snores more than half the time																
A2. Always snores																
A3. Snores loudly																
A4. Has deep or noisy breathing.																
A5. Are you having difficulty breathing or struggling to breathe?																
A6. Have you ever seen your son (or daughter) stop breathing during sleep?																
**Your son (or daughter):**																
A7. Tends to breathe with their mouth open during the day.																
A8. Wakes up with a dry mouth.																
A9. Wets the bed occasionally.																
**Your son (or daughter):**																
B1. Do you wake up tired in the morning?																
B2. Do you have trouble sleeping during the day?																
B3. Has a teacher or other person ever commented that your child seems sleepy during the day?																
B4. Is it difficult to wake your child in the morning?																
B5. Does your child wake up with a headache in the morning?																
B6. Did your child stop growing normally at any point since birth?																
B7. Is your child overweight?																
**Your son often:**																
C1. He doesn't seem to hear when spoken to directly.																
C2. Has difficulty organizing tasks and activities.																
C3. Is easily distracted by external stimuli.																
C4. Fidgets with hands or feet or becomes restless when seated.																
C5. Cannot sit still or often acts as if plugged into an electrical outlet.																
C6. Interrupts people or butts into conversations or games.																
TOTAL																
**Action to take after a positive PSQ (>** 9 "**Yes" responses): () Request polysomnography () Refer to a sleep specialist. () Clinical follow-up Notes:_____________________________________________________________________________________________________________________________________________________________________________________________________________________________________________________________________**																
**FOLLOW-UP ENT RECORD**																
**Patient's name:**																
**Medical Record Number: Date of Birth: / /**																
**Age: Sex: () F () M**																
**Name of the person in charge:**																
**Telephone:**																
**Time interval since last ENT evaluation**																
**Intervening events in the last year: () Hospitalizations () Surgeries () Recurrent infections**																
**Medical History Reports:______________________________________________________________________________________________________________________________________________________________________________________________________________________________________________________________________________________________________________________________________________________________**																
**TREATMENTS**																
	YES			NO										Observations		
Currently undergoing specific treatment																
Other treatments																
**GROWTH MILESTONES** Hoover-Fong et al.^49^																
	Measurement			Percentile										Observations		
Weight																
Head circumference																
Height																
Weight/Height																
**MOTOR DEVELOPMENT** Ireland et al.^50^																
	Age			Percentile										Observations		
Cervical control (months)																
Sat with support (months)																
Sat up without support (months)																
Crawled (months)																
Independent march (months)																
**FEEDING**																
without difficulty () with difficulty () Which ones:																
Observations (need for support, semi-sitting, hypotonia, acceptance of solid foods/tongue protrusion reflex, food refusal, cough, choking).																
**SPEECH AND LANGUAGE DEVELOPMENT** Ireland et al.^50^																
(Characteristics, form of communication and specific evaluations):																
	YES			NO						Observations						
Is he/she in school or daycare?										Stage?						
Learning difficulties																
Speech therapy																
**OTORHINOLARYNGOLOGICAL SIGNS AND SYMPTOMS**																
	YES			NO						Observations						
Nasal obstruction																
Respiratory noise																
Mouth breathing																
Snoring																
Tonsillitis																
Otitis										Otorrhea?						
Rhinosinusitis																
Hearing loss																
Salivary control																
Clinical suspicion of Obstructive Sleep Apnea (OSA)																
Dysphagia										() Liquid () Paste () Solid						
Choking										() Liquid () Paste () Solid						
Previous aspiration pneumonia																
Otorhinolaryngological Surgery										Which?						
**OTORHINOLARYNGOLOGICAL EXAMINATION**																
	YES			NO						Observations						
Oropharyngeal abnormalities																
Palate abnormalities																
Dental abnormalities																
Language abnormalities																
Anterior rhinoscopy																
Otoscopy																
Facial skeletal abnormalities (Midface hypoplasia, micrognathia, retrognathia)																
Skull base / possible narrowing of the foramen magnum (clinical suspicion)																
Other facial morphological abnormalities																
**ADDITIONAL TESTS IN OTORHINOLARYNGOLOGY**																
**Exam**	**Normal**			**Changed**						**Unrealized**						
Pure-tone audiometry																
Impedance audiometry																
Auditory Evoked Potentials																
Videonasopharyngolaryngoscopy																
Polysomnography																
**PSQ QUESTIONNAIRE PORTUGUESE VERSION – BRAZIL** Martins et al.^27^**APPLICATION DATE:**																
**While your child sleeps:**			**Yes**				**No**								**Don't know**	
A1. Snores more than half the time																
A2. Always snores																
A3. Snores loudly																
A4. Has deep or noisy breathing.																
A5. Are you having difficulty breathing or struggling to breathe?																
A6. Have you ever seen your son (or daughter) stop breathing during sleep?																
**Your son (or daughter):**																
A7. Tends to breathe with their mouth open during the day.																
A8. Wakes up with a dry mouth.																
A9. Wets the bed occasionally.																
**Your son (or daughter):**																
B1. Do you wake up tired in the morning?																
B2. Do you have trouble sleeping during the day?																
B3. Has a teacher or other person ever commented that your child seems sleepy during the day?																
B4. Is it difficult to wake your child in the morning?																
B5. Does your child wake up with a headache in the morning?																
B6. Did your child stop growing normally at any point since birth?																
B7. Is your child overweight?																
**Your son often:**																
C1. He doesn't seem to hear when spoken to directly.																
C2. Has difficulty organizing tasks and activities.																
C3. Is easily distracted by external stimuli.																
C4. Fidgets with hands or feet or becomes restless when seated.																
C5. Cannot sit still or often acts as if plugged into an electrical outlet.																
C6. Interrupts people or butts into conversations or games.																
TOTAL																
**Action to take after a positive PSQ (>** 9 "**Yes" responses): () Request polysomnography () Refer to a sleep specialist. () Clinical follow-up Notes:_________________________________________________________________________________________________________________________________________________________________________________________________________________________________________________________________________________**																

### Protocol pilot application

During the pilot phase, the protocol was applied to four male patients, with confirmed achondroplasia diagnosis, followed at the medical genetics outpatient clinic of the Hospital Universitário Júlio Muller, Universidade Federal de Mato Grosso (HUUFMT), aged 2- to 10-years, to assess feasibility, clarity, and applicability in clinical practice.

After the pilot application, no further modifications were required, and the protocol enabled the standardization of otorhinolaryngological care for patients with achondroplasia, encompassing the main otorhinolaryngological manifestations. It will facilitate clearer communication among healthcare professionals involved in their care and enable future research based on this standardization.

## Discussion

At revision, the predominance of studies in the pediatric population raises concerns about the transition of care throughout life for individuals with achondroplasia.

The absolute citation frequency for each otorhinolaryngological manifestation in achondroplasia in the 27 studies is similar to that observed in other studies not included in the review.^51^, ^52^, ^53^, ^54^

The results of the absolute citation frequency of each otorhinolaryngological manifestation per article, synthesized from 11 prevalence studies, confirmed the high prevalence of these manifestations in achondroplasia, with a predominance of respiratory and otological abnormalities.

The range of each otorhinolaryngological manifestation reported across the 11 prevalence studies was consistent with analyses of the full set of 27 studies, with sleep apnea and hearing loss ranking first in both analyses.

The findings are consistent with literature, reporting snoring prevalence from 25% to 95%^51^ and Obstructive Sleep Apnea (OSA) prevalence of approximately 56% in infants and 60% in adults;^51^ 69.3% across all age groups;^52^ and 40.7%.^53^ These values are markedly higher than those in the general adult population (16%)^55^ and the pediatric population (1%–5%).^56^

The literature reports that the prevalence of otitis media in achondroplasia ranges from 48% to 80% in children up to 5-years of age, 33% in adolescents, and 17% in adults,^51^ much higher than in the general population by 10%–11%.^57^ Hearing loss occurs in 55% of achondroplastic individuals regardless of age,^51^ with other cohorts reporting prevalences above 70%^52^ and 61.1%.^53^ These data highlight the need for systematic otorhinolaryngological follow-up in this population.

After expert panel validation, the result was 0.94, which indicates excellent agreement on relevance, clarity, and adequacy. The protocol pilot application proved to be feasible and easy to use in an outpatient setting, with a mean application time of 34 min. However, there is still a need to expand its use through application in other healthcare settings in order to test its feasibility in different contexts and to incorporate further improvements, increasing its robustness, and thereby enable early identification of otorhinolaryngological complications, guide appropriate therapeutic decision-making, improve the quality of care, and serve as a database for future research.

## Conclusions

The protocol developed in this study addressed fundamental aspects of clinical assessment, including gestational and neonatal history, developmental milestones, feeding, speech and language development, otorhinolaryngological signs and symptoms, specialized physical examination, and complementary tests, and incorporation of the Pediatric Sleep Questionnaire (PSQ) as a screening tool for sleep-disordered breathing.^27^

Content validation by an expert panel demonstrated high agreement regarding clarity, relevance, and applicability of the proposed items, improving the instrument. Additionally, the pilot application in patients followed at the Hospital Universitário Júlio Muller, Universidade Federal de Mato Grosso (HUUFMT) enabled its feasibility in clinical practice.

The proposed protocol is an evidence-based, feasible, and clinically applicable tool, that assists in standardized otorhinolaryngological assessment of patients with achondroplasia. Its implementation may contribute to the early complications identification, guide appropriate therapeutic decision, improve care quality, and provide a robust database for future research.

## ORCID ID

Cynthia Moreno: 0009-0004-3982-9390

Marcial Galera: 0000-0002-1624-7572

## Funding

This research has not received specific funding from public, commercial, or non-profit funding agencies.

## Data availability statement

The authors declare that all data are available in repository.

## Declaration of competing interest

The authors declare no conflicts of interest.
